# Canopy Nitrogen Concentration Monitoring Techniques of Summer Corn Based on Canopy Spectral Information

**DOI:** 10.3390/s19194123

**Published:** 2019-09-23

**Authors:** Lu Liu, Zhigong Peng, Baozhong Zhang, Zheng Wei, Nana Han, Shaozhe Lin, He Chen, Jiabing Cai

**Affiliations:** 1 State Key Laboratory of Simulation and Regulation of Water Cycle in River Basin, China Institute of Water Resources and Hydropower Research, Beijing 100038, China; 18801090061@163.com (L.L.); weizheng@iwhr.com (Z.W.); hnn23144@163.com (N.H.); 18763898993@163.com (S.L.); chenhe@iwhr.com (H.C.); caijb@iwhr.com (J.C.); 2 National Center of Efficient Irrigation Engineering and Technology Research-Beijing, Beijing 100048, China

**Keywords:** summer corn, hyperspectral, canopy nitrogen concentration, sensitive bands, spectral index model

## Abstract

Crop nitrogen monitoring techniques, particularly choosing sensitive monitoring bands and suitable monitoring models, have great significance both in theory and in practice for achieving non-destructive monitoring of nitrogen concentration and accurate management of water and fertilizer in large-scale areas. In this study, a lysimeter experiment was carried out to examine the characteristics of canopy spectral reflectance variation of summer corn under different fertilization levels. The relationship between canopy spectral reflectance and nitrogen concentration was investigated, based on which sensitive bands for the corn canopy nitrogen monitoring were selected and a suitable spectral index model was determined. The results suggest that under different fertilization levels, the canopy spectral reflectance of summer corn decreases with the increase of the canopy nitrogen concentration in the visible light band, but varies in the opposite direction in the near-infrared band, with a premium put on a higher correlation between the spectral reflectance of the characteristic bands and their first derivatives and the canopy nitrogen concentration. The most sensitive bands for monitoring the canopy nitrogen concentration using spectral reflectance and its first derivative are found to be 762 nm and 726 nm and the correlation coefficients are 0.550 and 0.795, respectively. The optimal band combination, generated by multivariate stepwise regression analysis, is composed of 762 nm, 944 nm and 957 nm bands. From the 55 reported spectral index models of crop nitrogen concentration monitoring, the most suitable index model, NDRE, is chosen such that this index model has the highest correlation with the canopy nitrogen concentration in summer corn. This model has a significant positive correlation with the canopy nitrogen concentration at each growth period, and the correlation coefficient is up to 0.738 during the whole growth period. Spectral monitoring models of canopy nitrogen concentration are constructed using sensitive bands, and a combination of bands and the spectral index, suggesting that these models perform well in monitoring. The models arranged in descending order of simulation accuracy are as follows: the suitable spectral index model, the optimal band combination model, the sensitive band reflectance first derivative model, the sensitive band reflectance model. The determination coefficients are 0.754, 0.711, 0.639 and 0.306, respectively.

## 1. Introduction

Corn is a widely grown food crop, and nitrogen fertilizer is one of the major limiting factors affecting its growth, playing an important role in growth, yield, and quality of corn. However, aimless increase in fertilizer application and low efficiency of use are having ever greater adverse effects [1]. In order to optimize the nitrogen management in the process of crop growth, it is necessary to understand the nitrogen status of crops accurately and in time [2]. Therefore, rapid but effective tracking and monitoring of crop nitrogen concentration situations, and then reasonable application of nitrogen fertilizer, are of great significance for improving corn quality and for sustainable land use. Crops show different spectral characteristics with different nitrogen concentrations, as has already confirmed by many studies [3]. Compared with traditional destructive sampling methods to monitor plant canopy nitrogen concentration, non-destructive methods that are able to acquire crop hyperspectral information over a large area are more convenient, straightforward and able to provide scientific support for accurate management of modern, large-scale agricultural water and fertilizer application.

Spectral technology allows quick, non-destructive monitoring of crop nutritional parameters. It is one of the important concentrations of agricultural remote sensing to analyze the spectral reflection characteristics of crop canopy and estimate the nitrogen status in the process of crop growth [4]. Many scholars have made similar studies on wheat [5,6,7], rice [8] and corn [9,10], all as field crops, and after analyzing the correlation between the nitrogen content of the plants and their spectral reflectance, built some spectral models for estimating the nitrogen content of the plants. Liu et al. point out that monitoring the nitrogen content of corn in the red and green light bands produces better results, though the sensitive bands vary from one growth period to another [1]. Wang et al. point out that the serious nitrogen deficiency, normal nitrogen application and excessive nitrogen application could be qualitatively distinguished by the spectral reflectivity curve of rice, which laid a foundation for the establishment of a rice canopy nitrogen nutrition diagnosis model in the future [11]. Zhao et al. think that the most sensitive bands were located at 710 nm and 512 nm [12]. Stroppiana et al. used two years of field experiments to propose a regression model for predicting nitrogen concentration in rice plants by spectral data; the model has good applicability through verification [13]. Regarding monitoring the nitrogen content of plants using a spectral index model, previous hyperspectral studies on N accumulation, (leaf area index) LAI and biomass of corn show that hyperspectral remote sensing can be used to comprehensively evaluate the growth of corn [14,15,16]. Clevers et al. note that the green chlorophyll index (CI green) and the red edge chlorophyll index (CI red-edge) are effective in monitoring the nitrogen content [17,18]; Hansen et al. point out that the normalized difference vegetation index (NDVI) and the double-peaked canopy nitrogen index (DCNI) can satisfactorily monitor the wheat canopy nitrogen content [19]. Rasooli et al. show that stepwise multiple linear regressions can be put to good use in constructing a fitted model of protein content in winter wheat leaf and grain [20]. In recent years, N nutrition index (NNI) has been considered to be a reliable index for crop diagnosis [21]. In summary, from the researches carried out on the quantitative relationship between the spectral reflectance of crops and the nitrogen concentration of their plants, it can be seen that large differences exist in both spectral extraction and analysis methods used in spectral remote sensing monitoring. Some of these studies are based on qualitative or semi-quantitative experiments but making use of sensitive bands or spectral index models to monitor the nitrogen concentration of plants is troubled by universality in applications. Moreover, as crops, growth period, and investigation region vary from study to study, the sensitive bands and spectral index models for monitoring plant nitrogen concentration vary from study to study too, making these models unable to meet the general needs of crop nutrition diagnosis and growth monitoring. In order to achieve large-scale, non-destructive monitoring of nitrogen concentration in main crop plants, it is urgent to carry out further spectral monitoring tests of nitrogen concentration in typical crops and to choose the most sensitive bands and spectral index models suitable for monitoring nitrogen concentration during the whole growth period, as an effort to solve the technical problem of universality associated with spectral characteristic variables.

The study reported in this paper focuses on summer corn. A hand-held ground spectrometer and a Kjeldahl nitrogen apparatus were used to investigate the spectral monitoring of canopy nitrogen concentration under different nitrogen fertilizer treatments. The sensitive bands and the suitable hyperspectral estimation models chosen may provide a scientific basis for hyperspectral diagnosis of nitrogen concentration in corn.

## 2. Materials and Methods

### 2.1. Brief Information of Experiment Area

The experiment was carried out at the water-saving irrigation experiment station of China Institute of Water Resources and Hydropower Research. The station is located at 39°37′N and 116°26′E, at an altitude of 40.1 m, dominated by warm and semi-arid continental monsoon climate, dry in winter and spring and rainy in summer. The mean annual precipitation is 540 mm. The mean annual temperature is 12.1 °C, and the mean annual wind speed is 1.2 m/s. The mean annual frost-free period is 185 d and the mean annual surface evaporation is above 1800 mm. The mean annual sunshine hours are about 2600 h.

### 2.2. Experiment Design

The summer corn variety studied was Jiyuan 168, sown on June 15, harvested on September 25 for 2017 and 2018 at plant spacing and row spacing both of 25 cm. The soil is sandy loam, and the initial nutrient status of the soil is shown in Table 1. The experiment involved a total of 12 lysimeters of 1 m × 0.75 m × 1 m size each, with six plants per box. The lysimeter experiment received four nitrogen fertilizer treatments and had two fertilizer applications throughout the growth period. More specifically, the base fertilizer was compound fertilizer (15% N, 15% P_2_O_5_, and 15% K_2_O), and during the shooting–tasseling period, urea was applied (46% N); the proportion of compound fertilizer to urea was 1:1. The setting of specific fertilizer level refers to the guidance of scientific fertilizer application of the main crops of the Ministry of Agriculture, and the gradient setting was carried out in combination with the local fertilizer level (450 kg/hm^2^) in the experimental site. The amount of fertilization was 0, 225, 450, 675 kg/hm^2^, that is, *N0*, *N1*, *N2*, and *N3*, and each treatment was repeated three times. Spectral monitoring for 2 years involved four growth periods. During the spectral determination period, three representative corn canopies in each box were selected for canopy spectral monitoring, the average values were used as the spectral reflectance of the box, and the average values of three boxes in the same treatment were used as the corn canopy spectral reflectance of the same treatment. Because the nitrogen concentration of corn canopy was determined as destructive sampling, only two representative plants were selected for the determination of nitrogen concentration for the same treatment. Other management practices such as sowing, farming, and weeding were kept in line with local farmers. Compared with the field plot experiment, the lysimeter experiment can strictly control the experimental variables, was subject to less interference and its data are more reliable.

### 2.3. Items to Be Measured and Methods

#### 2.3.1. Monitoring of Canopy Spectra

During the growth period of summer corn, the canopy spectral reflectance was monitored at 10:00−14:00 using a Field-Spec HandHeld2, a hand-held ground spectrometer manufactured by American Analytical Spectral Devices (ASD), on a fine day with still wind or light breeze. The band was 325−1075 nm, the sampling interval was 1 nm, with a resolution of 3 nm. During the measurement, the sensor probe was aimed vertically downward above the canopy, the field of view angle of the spectrometer was 25°, and the vertical height from the top of canopy was about 15−20 cm.

#### 2.3.2. Determination of Canopy Nitrogen Concentration

Young fully expanded leaves were sampled to determine canopy nitrogen concentration. Samples were cut from the plants and were immediately taken back to the laboratory for water-removing and oven-drying treatment. After drying, the sample was ground to a uniform powder, which was then boiled in H_2_SO_4_-H_2_O_2_ solution. Measurement was made with a Kjeldahl nitrogen apparatus. More details of this measurement method can be found in *Soil Agrochemical Analysis* [22].

#### 2.3.3. Data Processing and Statistical Analysis

The monitored raw spectral reflectance data were resampled and exported by the processing software of the spectrometer. The arithmetic mean of the spectral reflectance at all sampling points was taken as the raw spectral reflectance of the canopy. From these raw spectral reflectance data, the first derivative was obtained. Then, correlations between the nitrogen concentration of the summer corn canopy on the one hand and the raw spectral reflectance and the first-order differential spectrum on the other hand were estimated.

The correlation coefficient *r* described above was estimated by:

$$r=\frac{\sum_{i=1}^{n}\left(x_{i}-\bar{x})(y_{i}-\bar{y}\right)}{\sqrt{\sum_{i=1}^{n}{\left(x_{i}-\bar{x}\right)}^{2}•\sum_{i=1}^{n}{\left(y_{i}-\bar{y}\right)}^{2}}},$$

where *n* is the number of actual measurements; *x_i_* is the spectral reflectance or first-order differential spectrum of the *i*-th summer corn canopy; *x* is the mean of the spectral reflectance, or the mean of the first-order differential spectra of the summer corn canopy; *y_i_* is the nitrogen concentration in the *i*-th summer corn canopy; and *y* is the mean of summer corn canopy nitrogen concentration.

## 3. Results

### 3.1. Canopy Nitrogen Concentration and Yield of Summer Corn under Different Nitrogen Levels

**Figure 1:**
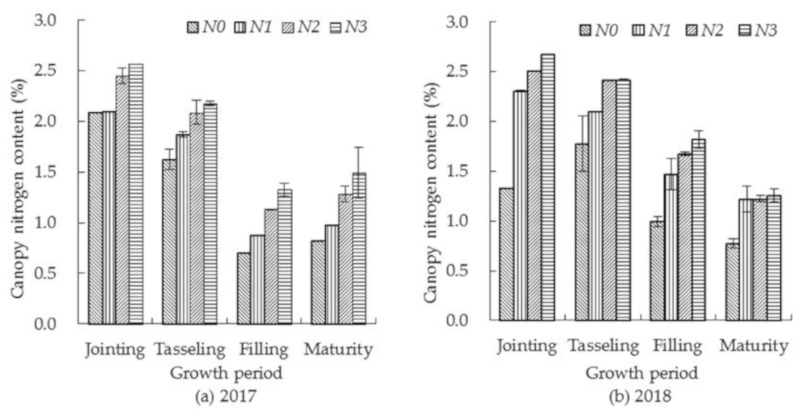
Effect of nitrogen application level on summer corn canopy nitrogen concentration.

The canopy nitrogen concentrations of summer corn plants under different nitrogen treatments are shown in Figure 1. The concentration dropped as the growth period progressed, being higher at the jointing and the tasseling periods and lower at the filling and the maturing periods. With the increase of nitrogen fertilization, the canopy nitrogen concentration of summer corn increased, with the concentration under different nitrogen fertilizer treatments differing to an extremely significant level (*p* < 0.01), but the difference between *N2* and *N3* treatments just reached a significant level (*p* < 0.05).

**Figure 2:**
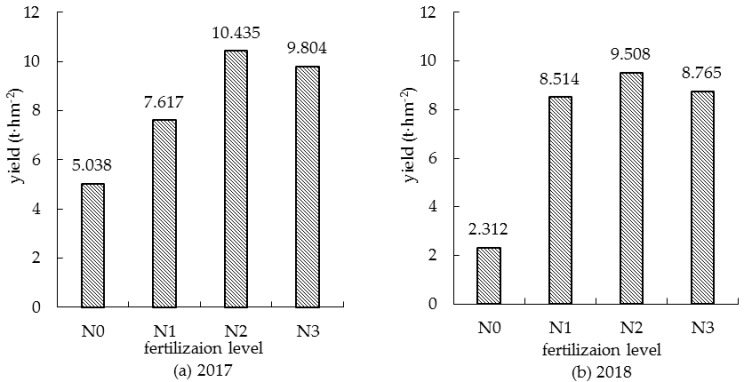
Effect of nitrogen application level on yield of summer corn.

The yields of summer corn under different nitrogen fertilizer treatment are shown in Figure 2. The yield of summer corn increased with the increase of nitrogen fertilization. The maximum yield was 10.435 t/hm^2^ for 2017 and 9.508 t/hm^2^ for 2018 in the case of treatment *N2*. As the application rate of the nitrogen fertilizer was further improved, the yield was decreased, as in the case of *N3* treatment. This demonstrates that rational fertilization promotes corn growth and ensures a high yield. Controlling surface source pollution caused by excessive fertilization, as can be seen, is conducive to sustainable and efficient agricultural production.

### 3.2. Canopy Spectral Characteristics of Summer Corn under Different Nitrogen Levels

**Figure 3:**
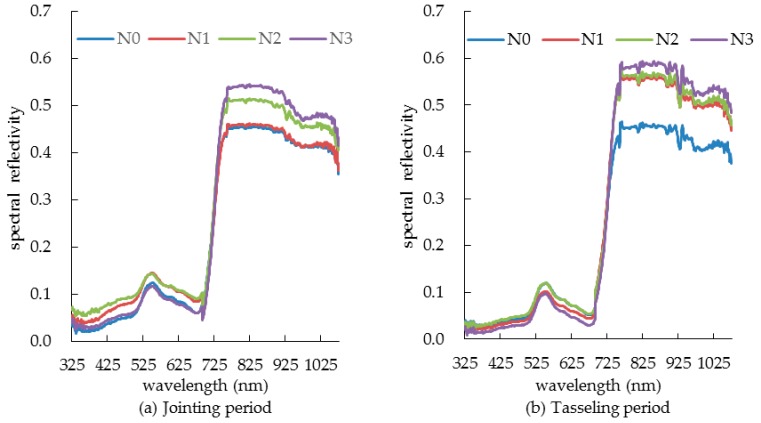

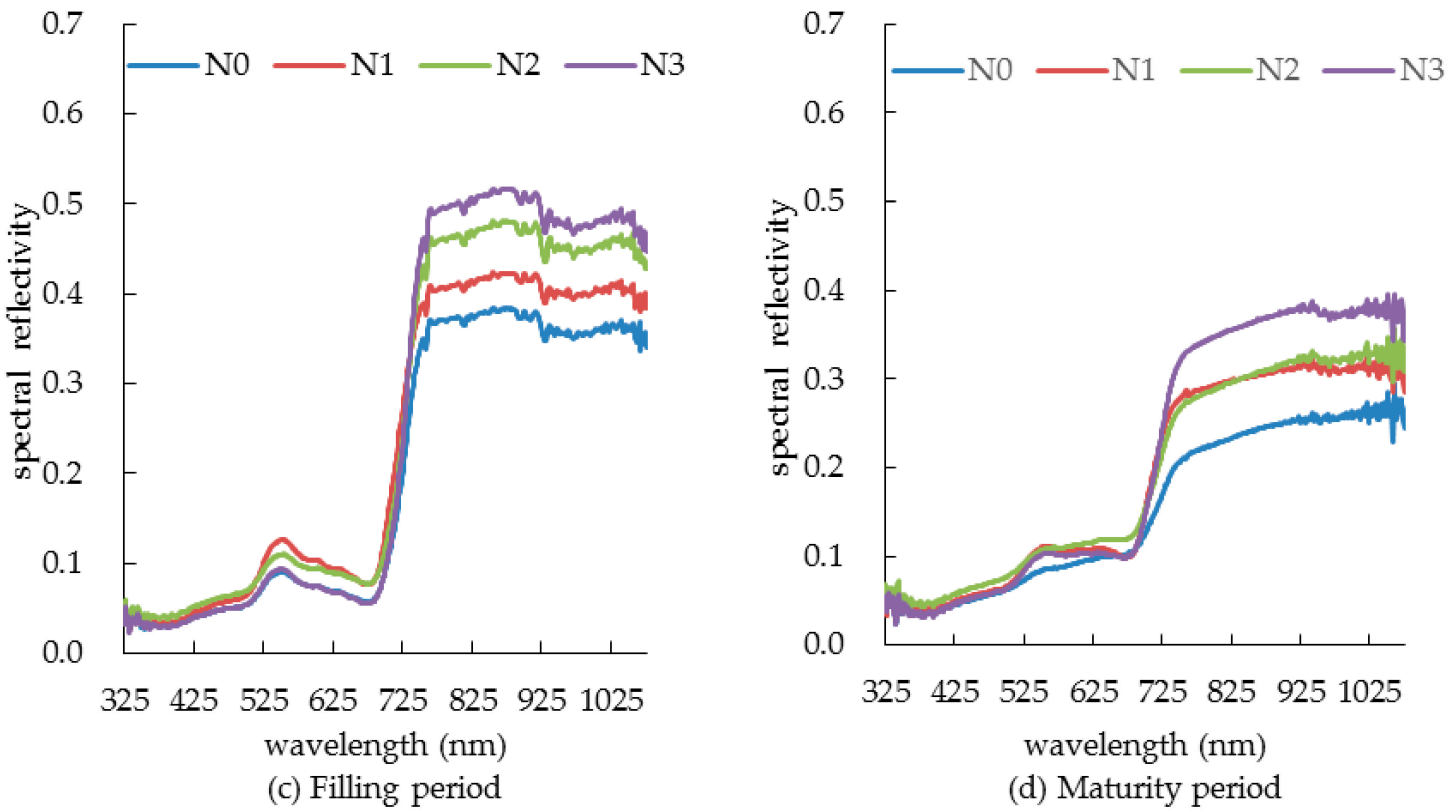
Effect of nitrogen application levels on canopy spectral reflectance curve of summer corn.

The canopy spectral characteristics of summer corn plants under different nitrogen treatments are shown in Figure 3. In the photosynthesis process, chlorophyll plays a central role in light absorption because in the visible light band (380−760 nm) chlorophyll absorbs most of the red and violet light but reflects green light, with the result that the canopy spectral reflectance is low. Specifically, the reflection peak of the green light band (500−580 nm) appears around 550 nm, while at the red light band (620−760 nm) an absorption band occurs because a large amount of red light is absorbed in the photosynthesis process of chlorophyll. In the near-infrared band (760−1075 nm), there occurs a high reflection zone because the canopy spectral reflectance is controlled by the internal structure of the summer corn canopy: the canopy reflectance rises sharply near the 760 nm band to form a “red edge”.

Under the same nitrogen treatments, during the growth process from jointing period to tasseling period, because summer corn was in the vigorous growth period, photosynthesis increased gradually with the increase of leaf area, and the coverage also increased, which effectively reduced the influence of soil background and other external factors, and the spectral reflectance of summer corn decreased gradually in the visible light band. From tasseling period to filling period, photosynthesis decreased gradually because of the decrease of chlorophyll concentration and the absorption capacity of blue and red light bands decreased; the spectral reflectance of summer corn canopy increased gradually. After entering the maturity period, the leaves withered yellow and the canopy spectral reflectance increased. While in the near infrared band, the canopy spectral reflectance increased gradually due to the jointing period and tasseling period belonging to the period of nutrient growth and accumulation under the control of internal structure of summer corn canopy. However, the filling period and maturity period are the reproductive growth period centered on grain, at the same time, the spectral reflectance of the canopy of summer corn was gradually decreased by the effect of the concentration of chlorophyll and the photosynthesis.

In the visible light band, the spectral reflectance of summer corn showed a clear difference under different nitrogen application levels, since chlorophyll plays a main role in the photosynthesis process and its concentration will have a direct influence on photosynthesis ability. However, the important component element of chlorophyll is nitrogen, so the SPAD value of high nitrogen application is larger than that of low nitrogen application [23], which affects the photosynthesis. Therefore, the spectral reflectance of summer corn canopy decreased with the increase of nitrogen application amount, among which there was significant difference (*p* < 0.05), especially in the green light band during the filling period, as shown in Figure 3c. In the range of near infrared band, summer corn was controlled by the internal structure of plant canopy and the variation rule of the canopy spectral reflectance of summer corn showed an opposite trend under different nitrogen application levels. With the increase of nitrogen application, the spectral reflectance of canopy increased, and the difference between treatments was more significant than that of the visible light band, especially at the filling period, and reached a very significant level (*p* < 0.01).

### 3.3. Sensitive Bands for Spectral Monitoring of Canopy Nitrogen in Summer Corn

**Figure 4:**
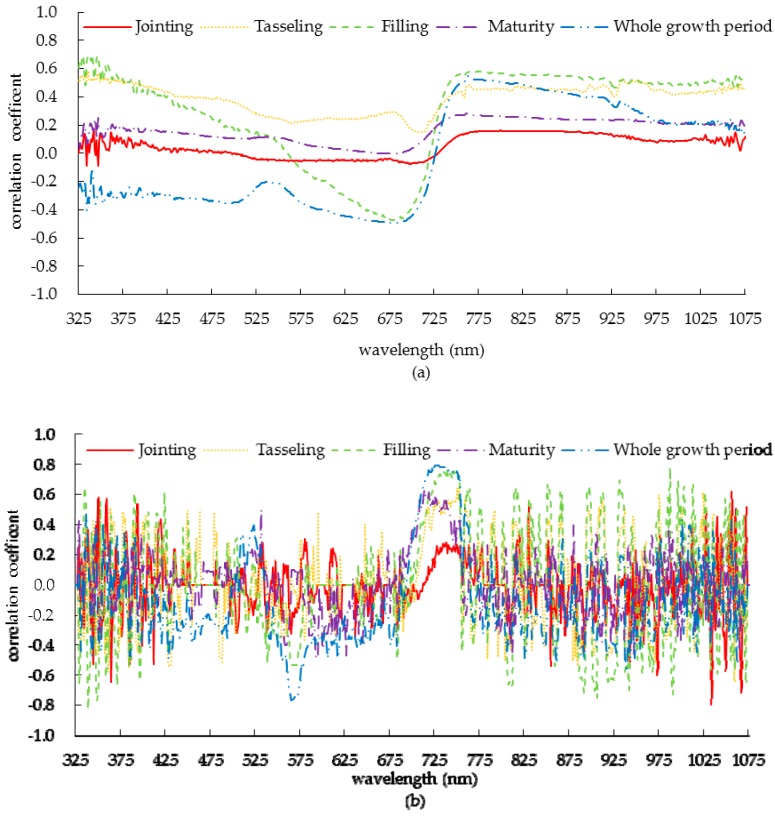
Correlation between canopy spectral reflectance and its first derivative and nitrogen concentration in summer corn. (**a**) the correlation coefficient between the canopy spectral reflectance and canopy nitrogen concentration of summer corn; (**b**) the correlation coefficient between the first derivative of the canopy spectral reflectance of summer corn and canopy nitrogen concentration.

The correlation coefficient between the canopy spectral reflectance and canopy nitrogen concentration of summer corn is illustrated in Figure 4a. It can be seen that the two are relatively low at the jointing period which may be due to the fact that the jointing period is in the peak period of vegetative growth, and the changes of nutrient composition and cell structure lead to the instability of spectral reflectance. There was a positive correlation between the canopy spectral reflectance and canopy nitrogen concentration at the tasseling period and the maturity period, which reached their maximums at 937 nm and 762 nm, which were 0.526 and 0.281, respectively. At the filling period, the correlation coefficients formed wave valley and wave peak at 680 nm and 774 nm, and the correlation coefficients were –0.474 and 0.478, respectively. Over the whole growth period, the correlation coefficients formed wave valleys and peaks at 678 nm and 762 nm and were –0.492 (*p* < 0.05) and 0.550 (*p* < 0.01), respectively.

The correlation coefficient between the first derivative of the canopy spectral reflectance of summer corn and canopy nitrogen concentration is shown in Figure 4b. By comparing with Figure 4a, it can be seen that the correlation coefficient between the first derivative value and the nitrogen concentration in some bands was higher than that between the spectral reflectance and the nitrogen concentration, which was due to the removal of the influence of soil background on the spectral reflectance in the derivation process. It can be seen that in 700−750 nm band, there was a significant positive correlation in the whole growth period, among which the correlation of filling period and whole growth period were the highest, followed by tasseling period and maturity period, and the lowest correlation was at jointing period.

**Figure 5:**
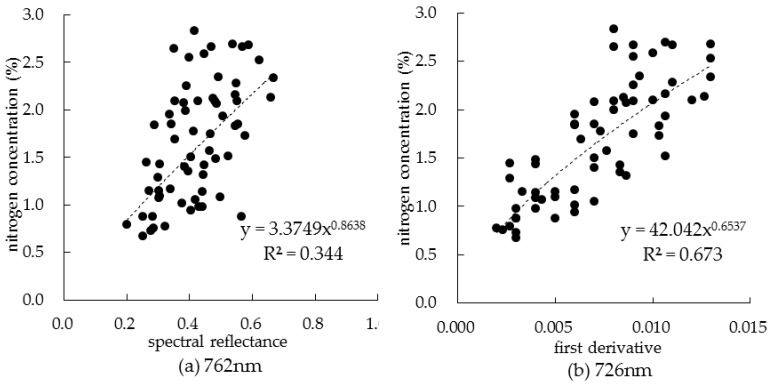
Fitting model of canopy nitrogen concentration and sensitive band.

According to the above analysis results, the most sensitive band was selected according to the principle of maximizing the correlation value, and the optimal band combination was selected by stepwise regression analysis. The sensitive bands based on the synthesis of two-year data are listed in Table 2. For the sensitive band screened by spectral reflectance, it can be seen that the correlation between spectral reflectance of sensitive band and nitrogen concentration was not significant at the jointing and maturity periods. At tasseling and filling periods, the reflectance of the sensitive band was significantly correlated with nitrogen concentration, and there was a very significant correlation at filling period. During the whole growth period, the correlation between them was very significant at 762 nm; the fitting model of canopy nitrogen concentration based on spectral reflectance is shown in Figure 5a. The results showed that the spectral reflectance of summer corn was easily affected by soil background and leaf structure at jointing period maturity periods compared with tasseling and filling periods, so it was difficult to obtain an ideal sensitive band.

For the first derivative of spectral reflectance, except for the jointing period, the correlation between the first derivative of spectral reflectance and the nitrogen concentration was very significant. For the sensitive band of 726 nm in the whole growth period, the first derivative of spectral reflectance had the highest correlation with nitrogen concentration, and the correlation coefficient was as high as 0.795. The fitting model of canopy nitrogen concentration based on first derivative of spectral reflectance is shown in Figure 5b. Obviously, the use of spectral reflectance first derivative selection was more stable compared to the use of spectral reflectance to select sensitive bands.

Based on synthesis of two-year data, the optimal band combination selected by stepwise regression analysis was 762 nm, 944 nm and 957 nm. The fitting model was y = 0.881 – 10.194R_762_ – 20.056R_957_ + 11.469R_944_, and the determination coefficient was as high as 0.711.

### 3.4. Selecting Optimal Index Model for Monitoring Canopy Nitrogen Concentration in Summer Corn

This study was based on 55 published nitrogen spectral monitoring index models (Table 3). The correlation between the calculated value of nitrogen spectral monitoring index and the monitoring value of nitrogen concentration based on synthesis of two-year data was analyzed and the correlation coefficients are listed in Table 4. 

**Figure 6:**
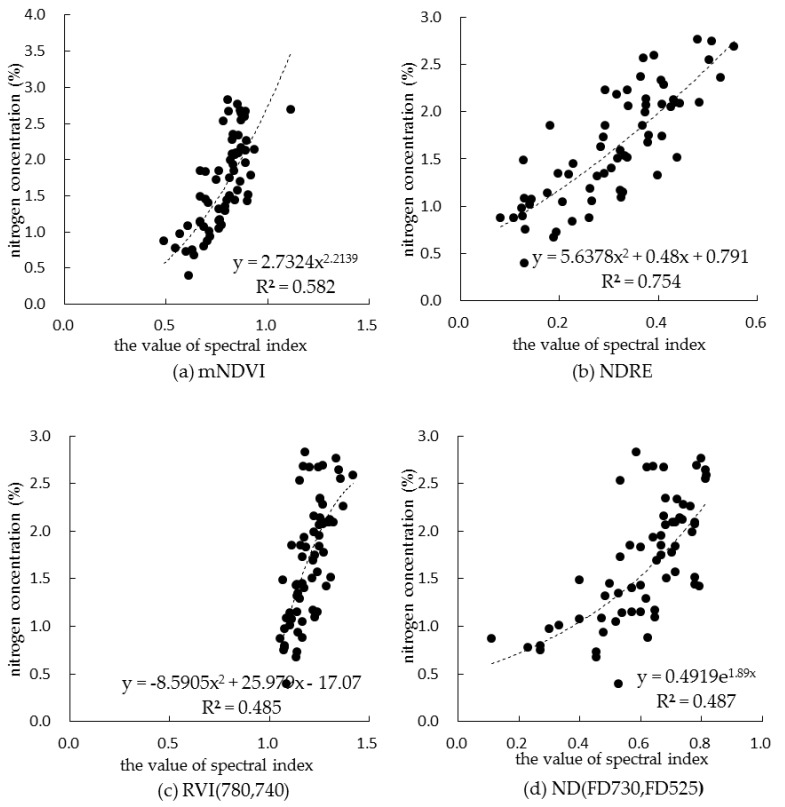

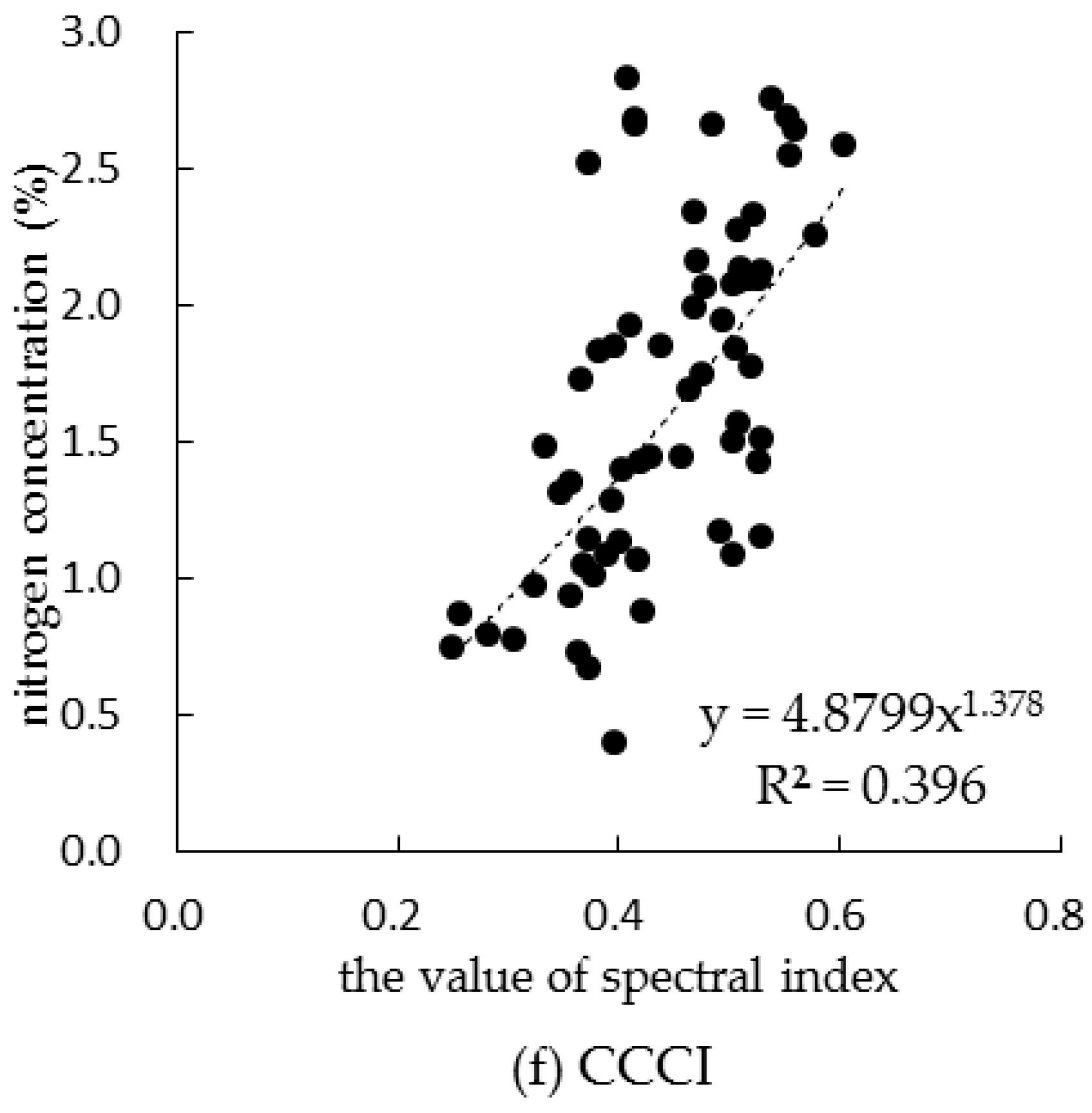
Fitting model of canopy nitrogen concentration based on top five spectral indexes.

The spectral index of monitoring canopy nitrogen concentration in summer corn during the whole growth period was selected based on two-years data, and the spectral indexes of the top 20 correlation coefficients were screened out as shown in Table 5. In order to enhance the applicability of the spectral index in monitoring canopy nitrogen concentration in each growth period of summer corn, the correlation coefficient between 20 spectral indexes and canopy nitrogen concentration in each growth period was considered synthetically, and finally the top five spectral indexes were selected as shown in Table 6; these were mNDVI, NDRE, R780/R740, ND (FD730, FD525) and CCCI. The fitting model of canopy nitrogen concentration based on the top five spectral indexes is shown in Figure 6.

The correlation coefficients between the five spectral indexes selected based on the comprehensive data of two years and the nitrogen concentration of the corresponding plant canopy were analyzed, as shown in Table 7 and Table 8. During the whole growth period, the spectral indexes reached very significant levels in 2017 and 2018, indicating that the spectral indexes had high interannual applicability. In 2017, the spectral indexes NDRE and R780/R740 were significantly correlated at all growth periods. In 2018, the five spectral indexes were significantly correlated at all growth periods. Considering the correlation between the spectral index and the nitrogen concentration of the plant canopy at each growth period, and combined with the difference of the nitrogen concentration of the canopy at different growth periods among different fertilizer treatments, NDRE was recommended as the most suitable monitoring model of the spectral index of nitrogen concentration in the whole growth period of summer corn.

### 3.5. Spectral Monitoring Technology of Canopy Nitrogen Concentration in Summer Corn

**Figure 7:**
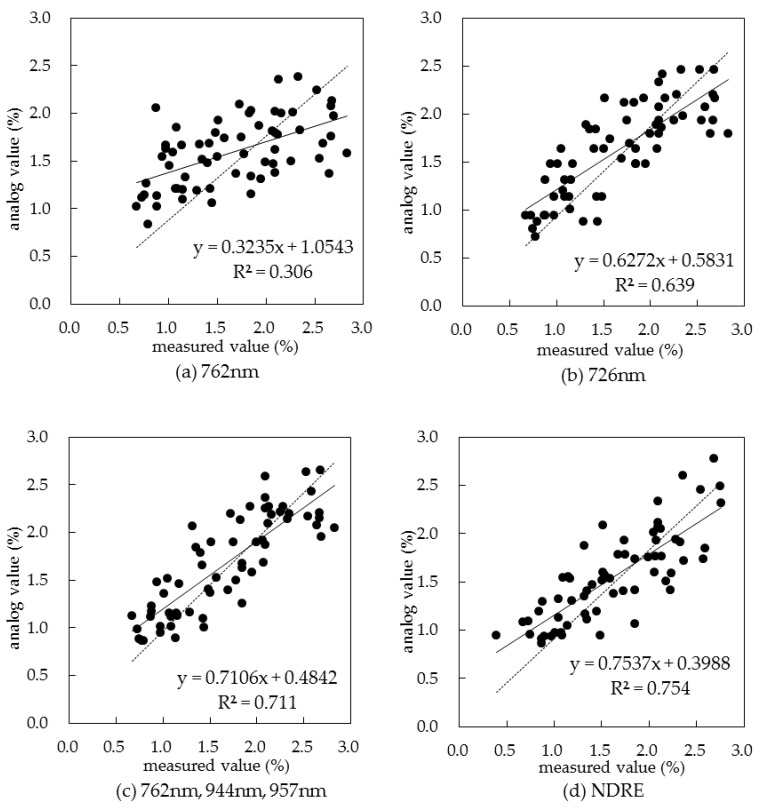
Comparisons between analog and measured values for the fitting models.

After the sensitive bands for the canopy nitrogen concentration monitoring were chosen and a suitable spectral index model recommended, we built a sensitive band reflectance model, a sensitive band reflectance first derivative model, an optimal bands combination model, and a suitable spectral index model (Table 9), obtained from the 762 nm spectral reflectance, the 726 nm spectral reflectance first derivative, the optimal bands combination obtained by stepwise discriminant analysis, and the spectral index NDRE. These models were compared for their monitoring performance in terms of determination coefficient (R^2^), root mean square error (RMSE), and mean absolute error (MAE), the specific meaning of which is given in [36], and the evaluation of the fitting model is shown in Figure 7. A monitoring model, incorporating the natural logarithmic function of the spectral reflectance of the sensitive 762 nm band, of summer corn canopy nitrogen concentration was established. So, this was a model based on the original spectral reflectance of the sensitive band. The R^2^, RMSE and MAE between the simulated values and the measured values were 0.306, 0.514 g.g^–1^, and 0.413 g.g^–1^ respectively. Another sensitive-band-based model, incorporating the natural logarithmic function of the spectral reflectance first derivative at the sensitive 726 nm band, was built for monitoring the canopy nitrogen concentration in summer corn. The R^2^, RMSE, and MAE between the simulated values and the measured values were 0.639, 0.368 g.g^–1^, and 0.298 g.g^–1^ respectively. A third model, based on the optimal combination of bands, was built by stepwise discriminant analysis. The R^2^, RMSE, and MAE between the simulated values and measured values were 0.711, 0.328 g.g^–1^, and 0.262g.g^–1^, respectively. A fourth model for monitoring the canopy nitrogen concentration in summer corn plants was based on the suitable spectral index model and incorporated a quadratic parabolic function of the calculated values of NDRE, the recommended suitable spectral index model. The R^2^, RMSE, and MAE between the simulated values and the measured values were 0.754, 0.322 g.g^–1^, and 0.258 g.g^–1^, respectively. It can be seen that the order of simulation accuracy from high to low was as follows: suitable spectral index model, band optimal combination model, sensitive band reflectivity first derivative model and sensitive band reflectivity model.

## 4. Discussion

It is very important to judge the nutritional status of crops accurately and in real time for achieving a high yield. In this study, the selection of spectral parameters such as sensitive band, spectral index and sensitive band combination are based on the simulation effect of the whole growth period as the evaluation standard. These can meet the requirements of spectral monitoring accuracy during the whole growth period of corn, break through the previous spectral model monitoring restricted by the growth period, and realize the accurate monitoring of nitrogen concentration during the whole growth period of corn. However, hyperspectral monitoring based on leaf scale could only represent the nitrogen concentration of a single plant and could only obtain hyperspectral reflectance in a small area, which is not representative. In order to facilitate crop nitrogen concentration monitoring in a large area, this needs to be achieved by means of remote sensing satellites in principle. However, most of the spectral reflectance obtained by satellite is crop canopy spectral reflectance. Therefore, it is very meaningful to study the spectral characteristics of crop canopy and establish the relationship between spectral reflectance and physiological and ecological parameters, which could be the basis of rapid diagnosis of crop nutritional status [37,38,39]. 

From the monitoring data of the canopy nitrogen concentration in summer corn and their canopy spectral reflectance data under different nitrogen levels, the spectral reflectance in the visible light band is mainly affected by the change of chlorophyll content. With the advance of growth period, the leaf area increases, while the soil background noise decreases. As a result, the spectral reflectance increases. In the near-infrared band, the spectral reflectance is mainly influenced by the optical properties and the canopy structure of the leaf structure; the multiple reflection and scattering of light inside the blade forms a high reflection platform, and the difference of cell gap, shape and composition also affect the spectral reflectance [26]. Therefore, the reflectance in the near infrared band decreases gradually with the advance of the growth period.

The sensitive bands and their suitable combination were put forward by analyzing the response relationship between the canopy nitrogen concentration and the canopy spectral reflectance. Compared with the original spectral reflectance, the correlation between the concentration of nitrogen in the canopy and the first derivative is higher, because the first derivative could reduce the influence of soil background and other noise. Osbome et al. pointed out that the sensitive band was in the green and red band for monitoring the canopy nitrogen concentration of the corn [40]. Li Zhen analyzed the nitrogen content in leaves and spectral reflectivity, which showed that the sensitive bands for monitoring nitrogen content were in blue light band and red light band [30]. This is consistent with our results, namely that the sensitive bands for monitoring nitrogen content are mainly in the red light band. Therefore, the sensitive band can be selected according to the change of spectral reflectance and its first derivative.

Because spectral reflectance is affected by many factors, spectral reflectance changes throughout the crop growth period. It is very difficult to obtain a simple and applicable spectral model for monitoring crop canopy nitrogen content. Four models for monitoring the canopy nitrogen concentration in summer corn, namely the sensitive band reflectance model, the sensitive band reflectance first derivative model, the suitable spectral index model, and the optimal band combination model were built based on the relationship between the spectral parameters and crop canopy nitrogen content for every growth stage. Compared with the regression model based on the sensitive band, the simulation precision based on spectral index and optimal band combination is higher. The spectral index and optimal band combination contain multiple band information, which can reduce the influence of soil background and other noise. The suitable spectral index model and the optimal spectral band combined regression model for monitoring the canopy nitrogen concentration in summer corn were proposed; these models had high correlation between the simulated values and the measured values for the whole growth period, which provides a reference for the spectral monitoring of nitrogen concentration in summer corn canopy.

## 5. Conclusions

The main conclusions are as follows:

(1) The canopy reflectance of the plants is low due to the absorption by chlorophyll in the visible light band, but the multi-scattering effect of the canopy cell structure in the near-infrared region leads to a higher reflectance in this band. At the point of fertilization, the canopy spectral reflectance of summer corn plants in the visible light band decreases with the increase of fertilization, but the trend is reversed in the near infrared band.
(2) Choosing the bands to which the plant canopy nitrogen concentration is sensitive reduces the redundancy of spectral information and improves the prediction accuracy of the spectral models. Investigation is made into the correlation between the summer corn plant canopy spectral reflectance and its first derivative on the one hand and the canopy nitrogen concentration on the other. From the correlation and factoring in the optimal band combination determined by the stepwise discriminant analysis, the sensitive bands for monitoring the canopy nitrogen concentration using the original spectra and their first derivative are found to be 762 nm and 726 nm respectively, the optimal combination of bands is 762 nm, 944 nm and 957 nm.
(3) A total of 55 published nitrogen spectral monitoring index models were examined for the correlation between their calculated values and the measured values of the canopy nitrogen concentration. Five spectral index models with higher correlation coefficients are retained, namely mNDVI, NDRE, R780/R740, ND (FD730, FD525) and CCCI, and the principle of highest correlation at key growth period was taken into account, NDRE is recommended as the most suitable spectral index model for monitoring nitrogen concentration in summer corn canopy.
(4) Once the sensitive bands were determined, the suitable spectral index model recommended, and the optimal band combination known, four models, namely the sensitive band reflectance model, the sensitive band reflectance first derivative model, the optimal band combination model, and the suitable spectral index model, were constructed and demonstrated to perform well in predicting summer corn canopy nitrogen concentration. The four models come in the following descending order of prediction accuracy: the suitable spectral index model, the optimal band combination model, the sensitive band reflectance first derivative model, and the sensitive band reflectance model.

## Figures and Tables

**Table 1:** Initial nutrient status of soil.

Degree of Depth (cm)	Initial Nitrate Nitrogen Concentration (mg/kg)	Initial Ammonium Nitrogen Concentration (mg/kg)
0−20	5.558	3.231
20−40	2.803	2.773
40−60	2.288	2.710
60−80	2.534	2.532

**Table 2:** Correlation coefficients between summer corn canopy nitrogen concentration and spectral reflectance and its first derivative.

Spectral Characteristic Variable		Jointing Period	Tasseling Period	Filling Period	Maturity Period	Whole Growth Period
spectral reflectance	Characteristic band (nm)	799	937	774	762	762
	Correlation coefficient	0.160	0.526 *	0.578 **	0.280	0.550 **
first derivative spectral reflectance	Characteristic band (nm)	737	752	738	714	726
	Correlation coefficient	0.285	0.659 **	0.767 **	0.636**	0.795 **

Note: ** *p* < 0.01, * *p* < 0.05.

**Table 3:** Indices used in the study for spectral monitoring of nitrogen.

Category	Spectral Parameters	Definition	Reference
Spectral characteristic parameters	Green peak amplitude, Rg	Maximum band reflectance within the green band of 510−560 nm	[5]
	Red trough amplitude, Rr	Minimum band reflectance within the red band of 640−680 nm	[5]
	(Rg – Rr) /(Rg + Rr)	Normalized value of green peak reflectance and red trough reflectance	[5]
	Rg/Rr	Ratio between green peak reflectance and red trough reflectance	[5]
	Red trough skewness, Sr	Band reflectance skewness within 640−680 nm region	[24]
	Red trough kurtosis, kr	Band reflectance kurtosis within 640−680 nm region	[24]
	Green peak skewness, Sg	Band reflectance skewness within 510−560 nm region	[24]
	Green peak kurtosis, kg	Band reflectance kurtosis within 510−560 nm region	[24]
	Sg/Sr	Ratio between green peak skewness (Sg) and red trough skewness (Sr)	[24]
	kg/kr	Ratio between green peak kurtosis (kg) and red trough kurtosis (kr)	[24]
	(Sg – Sr)/(Sg + Sr)	Normalized value of green peak skewness (Sg) and red trough skewness (Sr)	[24]
	(kg – kr)/(kg + kr)	Normalized value of green peak kurtosis (kg) and red trough kurtosis (kr)	[24]
	depth670	Vegetation absorption depth at 670 nm	[25]
	Area670	Vegetation absorption characteristic area at 560−760 nm, or the area between the envelope and the spectral reflectance in the spectral range of 560−760 nm.	[25]
	ND670	Normalized vegetation absorption depth at 670 nm, or the ratio between absorption depth and absorption characteristic area	[25]
	Red edge amplitude, Dr	Maximum first differential value of red edge in 680−760 nm region	[26]
	Blue edge amplitude, Db	Maximum first differential value of blue edge in 490−530 nm region	[26]
	Yellow edge amplitude, Dy	Maximum first differential value of yellow edge in 550−582 nm region	[26]
	Red edge area, SDr	Sum of first differential band values in the red edge waveband	[26]
	Blue edge area, SDb	Sum of first differential band values in the blue edge waveband	[26]
	Yellow edge area, SDy	Sum of first differential band values in the yellow edge waveband	[26]
	SDr/SDb	Ratio between the sum of first differential values in the red edge and that in the blue edge	[26]
	SDr/SDy	Ratio between the sum of first differential values in the red edge and that in the yellow edge	[26]
	SDr – SDb	Difference between the sum of first differential values in the red edge and that in the blue edge	[26]
	(SDr – SDb)/(SDr + SDb)	Normalized value of the sum of first differential values in the red edge and that in the blue edge	[26]
	(SDr – SDy)/(SDr + SDy)	Normalized value of the sum of first differential values in the red edge and that in the yellow edge	[26]
Spectral vegetation index	NPCI	(R430 − R680)/(R430 + R680)	[5]
	PRIb	(R570 − R539)/(R570 + R539)	[5]
	Soil adjustment vegetation index, SAVI	1.5 × (R870 − R680)/(R870 + R680 + 0.5)	[5]
	RVI (950, 660)	R950/R660	[6]
	RVI (810, 660)	R810/R660	[6]
	NRI	R800/R550	[9]
	RVI (810, 560)	R810/R560	[9]
	DCNI	(R720 − R700)/(R700 − R670)/(R720 − R670 + 0.03)	[9]
	MSR sum	(RNIR/RRED – 1)/(RNIR/RRED + 1)^0.5	[27]
		RNIR/RRED: ratio between sum of reflectance values in the near-infrared band (700−1075 nm) and that in the red light band (620−750 nm)	
	MSR mean	(RNIR/RRED – 1)/(RNIR/RRED + 1)^0.5	[27]
		RNIR/RRED: ratio between mean of reflectance values in the near-infrared band (700−1075nm) and that in the red light band (620−750 nm)	
	ND (FD730 , FD525)	(R′730 − R′525)/(R′730 + R′525)	[28]
	ND (573, 440)	(R573 − R440)/(R573 + R440)	[28]
	R810 – R680	R810 − R680	[28]
	RVI (780, 740)	R780/R740	[29]
	RVI (760, 510)	R760/R510	[30]
	RVI (760, 460)	R760/R460	[30]
	ND (760, 510)	(R760 − R510)/(R760 + R510)	[30]
	ND (740, 460)	(R740 − R460)/(R740 + R460)	[30]
	RSI (FD691 , FD711)	RSI(FD691, FD711) = R′691/R′711	[31]
	CCCI	((R780 − R720)/(R780 + R720)) /((R780 − R670)/(R780 + R670))	[32]
	NDRE	(R780 − R720)/(R780 + R720)	[32]
	mNDVI	(R816 − R732 × R537)/(R816 + R732 + R537)	[33]
	BNI	R434/(R496 + R401)	[33]
	mNDVI	(R924 − R703 + 2 × R423)/(R924 + R703 – 2 × R423)	[33]
	R′729	R′729	[34]
	RNIR – RRED MAX	Difference between maximum reflectance value in the near-infrared band (700−1075 nm) and that in the red light band (620−750 nm)	[35]
	RNIR – RRED MIN	Difference between minimum reflectance value in the near-infrared band (700−1075 nm) and that in the red light band (620−750 nm)	[35]
	RNIR – RRED sum	Difference between sum of reflectance values in the near-infrared band (700−1075 nm) and that in the red light band (620−750 nm)	[35]
	RNIR – RRED mean	Difference between mean of reflectance values in the near-infrared band (700−1075 nm) and that in the red light band (620−750 nm)	[35]

**Table 4:** Correlation coefficients between summer corn canopy nitrogen concentration and spectral index (two years).

	Spectral Index	Whole Growth Period	Jointing Period	Tasseling Period	Filling Period	Maturity Period
1	Green peak amplitude, Rg	−0.344	−0.509	0.345	0.025	−0.167
2	Red trough amplitude, Rr	−0.542	−0.421	0.533	−0.407	−0.167
3	(Rg – Rr)/(Rg + Rr)	0.743	0.004	−0.680	0.460	0.054
4	Rg/Rr	0.716	0.026	−0.668	0.437	0.043
5	Red trough skewness, Sr	0.087	0.278	0.075	0.355	0.333
6	Red trough kurtosis, kr	−0.243	0.242	0.023	−0.066	0.297
7	Green peak skewness, Sg	−0.407	−0.285	−0.236	−0.669	0.535
8	Green peak kurtosis, kg	0.534	0.301	0.257	0.549	−0.574
9	Sg/Sr	−0.078	0.206	−0.134	−0.131	0.389
10	kg/kr	0.398	−0.082	0.213	0.215	−0.443
11	(Sg – Sr)/(Sg + Sr)	0.283	−0.251	−0.118	−0.107	0.016
12	(kg – kr)/(kg + kr)	0.355	−0.087	0.215	0.184	−0.468
13	depth670	0.701	0.325	−0.122	0.529	0.286
14	Area670	−0.349	−0.506	0.414	0.031	−0.145
15	ND670	0.603	0.378	−0.264	0.544	0.268
16	Red edge amplitude, Dr	0.498	−0.300	0.557	0.542	0.035
17	Blue edge amplitude, Db	0.167	−0.434	0.065	0.086	−0.229
18	Yellow edge amplitude, Dy	−0.571	0.565	0.231	−0.357	0.140
19	Red edge area, SDr	0.706	−0.290	0.471	0.479	0.231
20	Blue edge area, SDb	0.044	−0.514	−0.145	−0.074	−0.236
21	Yellow edge area, SDy	−0.612	0.481	−0.237	−0.348	−0.244
22	SDr/SDb	0.605	0.370	0.621	0.638	0.516
23	SDr/SDy	0.108	−0.380	−0.045	0.526	0.016
24	SDr – SDb	0.741	−0.181	0.527	0.520	0.353
25	(SDr – SDb)/(SDr + SDb)	0.608	0.419	0.602	0.678	0.538
26	(SDr – SDy)/(SDr + SDy)	0.612	−0.429	−0.106	0.306	0.275
27	NPCI	0.773	−0.149	0.740	0.734	0.313
28	PRIb	−0.782	−0.321	−0.399	−0.563	−0.243
29	Soil adjustment vegetation index, SAVI	0.762	−0.098	0.410	0.523	0.327
30	RVI (950,660)	0.694	0.314	−0.028	0.496	0.293
31	RVI (810,660)	0.693	0.313	−0.135	0.519	0.292
32	NRI = R800/R550	0.612	0.368	0.188	0.496	0.423
33	RVI (810, 560)	0.611	0.371	0.259	0.514	0.412
34	DCNI	0.453	0.420	0.293	0.330	0.425
35	MSR sum	0.754	0.359	0.225	0.615	0.425
36	MSR mean	0.754	0.359	0.226	0.615	0.424
37	ND (FD730,FD525)	0.680	0.477	0.624	0.682	0.543
38	ND (573, 440)	0.195	−0.052	−0.810	−0.892	−0.453
39	R810 – R680	0.613	−0.255	0.473	0.476	0.205
40	RVI (780, 740)	0.687	0.372	0.637	0.718	0.522
41	RVI (760, 510)	0.611	0.328	−0.283	0.346	0.248
42	RVI (760, 460)	0.604	0.318	−0.474	0.175	0.203
43	ND (760, 510)	0.702	0.315	−0.339	0.372	0.286
44	ND (740, 460)	0.610	0.266	−0.603	0.013	0.170
45	RSI (FD691, FD711)	−0.612	−0.586	−0.409	−0.539	−0.478
46	CCCI	0.615	0.412	0.694	0.703	0.547
47	NDRE	0.771	0.390	0.524	0.735	0.569
48	mNDVI = (R816 – R732 – R537) /(R816 + R732 + R537)	0.704	0.384	0.444	0.590	0.468
49	BNI	0.402	0.018	−0.113	0.569	0.145
50	mNDVI = (R924 – R703 + 2 × R423) (R924 + R703 – 2 × R423)	0.738	0.451	0.799	0.864	0.538
51	R′729	0.767	−0.031	0.593	0.607	0.458
52	RNIR – RRED MAX	−0.402	0.276	0.609	0.039	0.080
53	RNIR – RRED MIN	−0.297	−0.512	−0.241	−0.176	−0.279
54	RNIR – RRED sum	0.352	−0.342	0.498	0.420	0.029
55	RNIR – RRED mean	0.606	−0.181	0.511	0.485	0.226

**Table 5:** The top 20 spectral indexes in each growth period.

Ranking	Whole Growth Period	Jointing Period	Tasseling Period	Filling Period	Maturity Period
1	PRIb	RSI(FD691,FD711)	ND(573,440)	ND(573,440)	kg
2	NPCI	Dy	mNDVI-1	mNDVI-1	NDRE
3	NDRE	SDb	NPCI	NDRE	CCCI
4	R′729	RNIR-RRED MIN	CCCI	NPCI	ND (FD730, FD525)
5	SAVI	Rg	(Rg − Rr)/(Rg + Rr)	RVI(780,740)	(SDr − SDb) /(SDr + SDb)
6	MSR mean	Area670	Rg/Rr	CCCI	mNDVI-1
7	MSR sum	SDy	RVI(780,740)	ND (FD730, FD525)	Sg
8	(Rg − Rr) /(Rg + Rr)	ND (FD730,FD525)	ND (FD730 ,FD525)	(SDr − SDb) /(SDr + SDb)	RVI (780, 740)
9	SDr − SDb	mNDVI-1	SDr/SDb	Sg	SDr/SDb
10	mNDVI-1	Db	RNIR-RRED MAX	SDr/SDb	RSI (FD691, FD711)
11	Rg/Rr	(SDr − SDy)/(SDr + SDy)	ND (740,460)	MSR mean	mNDVI-2
12	SDr	Rr	(SDr − SDb) /(SDr + SDb)	MSR sum	(kg − kr)/(kg + kr)
13	mNDVI-2	DCNI	R′729	R′729	R′729
14	ND (760, 510)	(SDr – SDb)/(SDr + SDb)	Dr	mNDVI-2	ND (573, 440)
15	depth670	CCCI	Rr	BNI	kg/kr
16	RVI (950, 660)	NDRE	SDr − SDb	PRIb	DCNI
17	RVI (810, 660)	mNDVI-2	NDRE	kg	MSR sum
18	RVI (780, 740)	SDr/SDy	RNIR-RRED mean	ND670	MSR mean
19	ND (FD730, FD525)	ND670	RNIR-RRED sum	Dr	NRI = R800/R550
20	CCCI	RVI (780, 740)	RVI (760, 460)	RSI (FD691, FD711)	RVI (810, 560)

Note: mNDVI-1 = (R924 – R703 + 2 × R423)/(R924 + R703 – 2 × R423); mNDVI-2 = (R816 – R732 – R537)/(R816 + R732 + R537).

**Table 6:** Correlation coefficients between corn canopy nitrogen concentration and spectral indexes (two years).

Spectral Index	Whole Growth Period	Jointing Period	Tasseling Period	Filling Period	Maturity Period
mNDVI = (R924 − R703 + 2 × R423) /(R924 + R703 − 2 × R423)	0.771 ^**^	0.451 ^*^	0.799 ^**^	0.864 ^**^	0.569 ^**^
NDRE = (R780 − R720)/(R780 + R720)	0.738 ^**^	0.390	0.524 ^*^	0.735 ^**^	0.538 ^**^
R780/R740	0.687 ^**^	0.372	0.637 ^**^	0.718 ^**^	0.522 ^**^
ND(FD730, FD525) = (R′730 − R′525)/(R′730 + R′525)	0.680 ^**^	0.477 ^*^	0.624 ^**^	0.682 ^**^	0.543 ^**^
CCCI = ((R780 − R720)/(R780 + R720))/ ((R780 − R670)/(R780 + R670))	0.615 ^**^	0.412	0.694 ^**^	0.703 ^**^	0.547 ^**^

Note: ** *p* < 0.01, * *p* < 0.05.

**Table 7:** Correlation coefficients between corn canopy nitrogen concentration and spectral indices (2017).

Spectral Index	Whole Growth Period	Jointing Period	Tasseling Period	Filling Period	Maturity Period
mNDVI = (R924 − R703 + 2 × R423) /(R924 + R703 − 2 × R423)	0.849 ^**^	0.319	0.821^**^	0.744^**^	0.589^**^
NDRE = (R780 − R720)/(R780 + R720)	0.856 ^**^	0.550 ^**^	0.692 ^**^	0.583 ^**^	0.584 ^**^
R780/R740	0.824 ^**^	0.579 ^**^	0.714 ^**^	0.591 ^**^	0.436 ^*^
ND (FD730, FD525) = (R′730 − R′525)/(R′730 + R′525)	0.778 ^**^	0.831 ^**^	0.799 ^**^	0.548 ^**^	0.328
CCCI = ((R780 − R720)/(R780 + R720))/ ((R780 – R670)/(R780 + R670))	0.762 ^**^	0.548 ^**^	0.746 ^**^	0.761 ^**^	0.363

Note: ** *p* < 0.01, * *p* < 0.05.

**Table 8:** Correlation coefficients between corn canopy nitrogen concentration and spectral indices (2018).

Spectral Index	Whole Growth Period	Jointing Period	Tasseling Period	Filling Period	Maturity Period
mNDVI = (R924 − R703 + 2 × R423) /(R924 + R703 − 2 × R423)	0.618 ^**^	0.509 ^*^	0.818 ^**^	0.855 ^**^	0.884 ^**^
NDRE = (R780 − R720)/(R780 + R720)	0.696 ^**^	0.470 ^*^	0.600 ^**^	0.673 ^**^	0.808 ^**^
R780/R740	0.543 ^**^	0.477 ^*^	0.597 ^**^	0.662 ^**^	0.904 ^**^
ND (FD730, FD525) = (R′730 − R′525)/(R′730 + R′525)	0.566 ^**^	0.563 ^**^	0.721 ^**^	0.636 ^**^	0.936 ^**^
CCCI = ((R780 − R720)/(R780 + R720))/ ((R780 − R670)/(R780 + R670))	0.511 ^*^	0.612 ^**^	0.755 ^**^	0.774 ^**^	0.884 ^**^

Note: ** *p* < 0.01, * *p* < 0.05.

**Table 9:** Evaluation of indexes of the fitting models during the whole growth period.

Spectral Parameters	Fitted Model	Model Evaluation Indexes		
		R^2^	RMSE (g.g^–1^)	MAE (g.g^–1^)
762 nm	y = 3.3749R_762_^0.8638^	0.306	0.514	0.413
726 nm	y = 42.042 (R_726_′)^0.6537^	0.639	0.368	0.298
762 nm, 944 nm, 957 nm	y = 0.881 − 10.194R_762_ − 20.056R_957_ + 11.469R_944_	0.711	0.328	0.262
NDRE	y = 5.6378x^2^ + 0.48x + 0.791	0.754	0.322	0.258
